# Techniques for identification of left ventricular asynchrony for cardiac resynchronization therapy in heart failure

**Published:** 2005-07-01

**Authors:** Peter Schuster, Svein Faerestrand

**Affiliations:** Department of Heart Disease, Haukeland University Hospital, Institute of Medicine, University of Bergen, Bergen, Norway

**Keywords:** cardiac resynchronization pacemaker therapy, electrical and mechanical asynchrony, echocardiography

## Abstract

The most recent treatment option of medically refractory heart failure includes cardiac resynchronization therapy (CRT) by biventricular pacing in selected patients in NYHA functional class III or IV heart failure. The widely used marker to indicate left ventricular (LV) asynchrony has been the surface ECG, but seems not to be a sufficient marker of the mechanical events within the LV and prediction of clinical response. This review presents an overview of techniques for identification of left ventricular intra- and interventricular asynchrony. Both manuscripts for electrical and mechanical asynchrony are reviewed, partly predicting response to CRT. In summary there is still no gold standard for assessment of LV asynchrony for CRT, but both traditional and new echocardiographic methods have shown asynchronous LV contraction in heart failure patients, and resynchronized LV contraction during CRT and should be implemented as additional methods for selecting patients to CRT.

## Introduction

### Cardiac Resynchronization Therapy

The most recent treatment option of medically refractory heart failure includes cardiac resynchronization therapy (CRT) by biventricular pacing in selected patients in NYHA functional class III or IV heart failure (HF) caused by idiopathic dilated (DCM) or ischemic cardiomyopathy, with QRS duration ≥ 130 milliseconds, left ventricular (LV) end-diastolic diameter ≥ 55 mm, and ejection fraction ≤ 35% [1].  Pacemaker treatment for severe HF started at the beginning of the 1980’s, when beneficial effects of dual chamber pacing with short atrioventricular-delay were claimed [2]. However, a later study did not confirm these results [3]. Modification of ventricular mechanical activation sequence by pacing from the right ventricular outflow tract also demonstrated various hemodynamic results [4-6]. Due to the fact that LBBB can cause asynchronous electrical activation and deterioration of LV pump function which can be corrected for by CRT,  a new era of CRT by biventricular pacing of both right and left ventricle has rapidly developed in recent years [7-9]. There are a large number of HF patients suffering from intra- and interventricular asynchronous contraction and relaxation assumed by the surface ECG  further deteriorating  an already hemodynamically compromised left ventricle [10]. Biventricular pacing is assumed to provide a more coordinated pattern of ventricular contraction, and reduce intraventricular and interventricular asynchrony.

The acute improvement of LV performance by CRT was demonstrated by reduction of capillary wedge pressure and an improvement in peak dp/dt without increase in myocardial oxygen consumption [11-14]. Long-term clinical benefits by CRT in terms of improvements in the 6-minutes hall walk distance, NYHA functional class and quality of life indices and increasing maximal and submaximal exercise capacity measured by oxygen consumption were demonstrated in several studies, even at diminished myocardial energy cost [15]. Even if CRT has proven to improve several hemodynamic and clinical indices in almost 70% of the patients, it is still difficult to define responders to CRT treatment, and most commonly composite clinical endpoints have been used. Of major interest is of course to decrease the number of clinical non responders, which is described to be at approximately 30 % [16-17].

The main focus of this manuscript is to review LV asynchrony, and to present an overview of techniques for identification of left ventricular intraventricular and interventricular asynchrony.

## Assessment of left ventricular asynchrony

### Electrical asynchrony

#### Surface ECG

The widely used marker to indicate LV asynchrony has been the surface ECG. The rational for that, is that the electrical delayed stimulation may lead to a mechanical delay of the respective LV areas. In LBBB the lateral free wall is activated later than the interventricular septum and thus leads to a delayed contraction of LV free wall. This surrogate marker represented by LBBB is one of the criteria indicating implantation of CRT [1]. It is shown that LBBB leads to impaired pump function  which can be improved by CRT [7-9].  LBBB is also a predictor of sudden cardiac death in DCM [18].

In 29 patients Alonso et al showed that reducing the QRS duration by CRT correlated to a positive clinical response suggesting a hemodynamic improvement associated with narrowing of QRS indicating reduction of the mechanical asynchrony of LV contraction. The study concluded even that the optimal placement for hemodynamic LV improvement of the right and LV leads would be those sites that could induce the greatest shortening of QRS duration [19]. However, still there is no evidence for prediction of clinical response based on baseline QRS width or by the reduction of the QRS width effected by CRT in the individual patient.

#### Electrophysiological mapping

An additional method to elucidate the intraventricular activation pattern in HF patients eligible for CRT is electroanatomical activation mapping.  In 26 patients Peichl et al. showed that the surface ECG is of limited value to describe the complex conduction disturbance of the LV [20]. They found differences in electroanatomical LV activation between ischemic cardiomyopathy and DCM with similar QRS morphology on the surface ECG.

Also Yu et al. showed that endocardial electrical LV activation sequences was variable among 7 HF patients with LBBB [21]. Lambiase et al. showed hemodynamic improvements in 10  HF patients by CRT in terms of increased cardiac output and dP/dt(max) when the LV was paced by placing the pacing lead away from areas  with demonstrated slow conduction [22]. They concluded that clinical non response may reflect LV lead placement in regions with slow conduction which can be avoided by pacing in more normally activated LV regions.

### Mechanical asynchrony

#### 3D-tagged magnetic resonance imaging (MRI)

Asynchronous contraction of LV was demonstrated in patients with DCM with intraventricular conduction delay by Curry et al., who used MRI [23]. MRI is a time consuming method and can so far not be employed in patients with implanted pacemakers.

#### Scintigraphic blood pool and phase image analysis

 By using gated equilibrium radionuclide angiography and Fourier phase analyses Fauchier et al. demonstrated in 103 patients  with DCM that QRS duration was related to both interventricular and intraventricular asynchrony [24]. Further, intraventricular asynchrony was an independent predictor of cardiac event (cardiac death, worsening of HF and heart transplantation) in DCM and the prognosis was related to intraventricular rather than to interventricular asynchrony.

Another radionuclide angioscintigraphy study by Toussaint JF et al.  in 21 patients showed that resynchronization by CRT between LV apex and base which persisted up to 12 months was also associated with a persisting improvement in LV systolic function [25]. The same group  showed in 34 patients that basal asynchrony and early resynchronization demonstrated by radionuclide angioscintigraphy might  predict long-term evolution of ventricular function after CRT in patients with very broad QRS (179 ± 18) [26].

Kerwin et al. used gated blood pool scintigraphy in 13 patients to demonstrate asynchronous contraction of LV and to show the resynchronizing effect of CRT [27]. DCM with intraventricular conduction delay was also associated with significant interventricular asynchrony. Improvements in interventricular synchrony during CRT correlated with acute improvements in LV ejection fraction. One limitation of the scintigraphic methods is the relatively poor time resolution of 30 fps.

#### Echocardiographic methods

Echocardiography is an essential method to diagnose HF and is also used to evaluate the effect of treatment to improve cardiac performance. The echocardiographic measurement of LV fractional shortening by M-mode and EF measured from two-dimensional images by using Simpsons method in accordance with the recommendations of the American Society of Echocardiography Committee, are widely used parameters for cardiac evaluation [28]. An additional method is the use of the Doppler principle to measure and quantify blood flow velocity. The method is based on the fact that the frequency of reflected ultrasound from a moving target towards the transducer is higher than the transmitted frequency.  When the reflecting target is moving away from the transducer, the frequency is lower than the original transmitted ultrasound frequency. The Doppler signals from the moving red blood cells have low amplitude and high velocity, and the echocardiographic methods used are continuous wave Doppler, pulsed Doppler and color flow imaging. A recently developed ultrasonographic method is tissue velocity imaging (TVI). This method uses frequency shifts of ultrasound waves to calculate myocardial tissue velocity at lower velocities, but higher intensity compared to blood flow velocity measurement. TVI can be used to measure movements of cardiac structures and to assess regional LV contractility, which has been shown in both animal experiments and human studies [29,30]. The TVI method is used in a wide range of cardiac diseases to characterize systolic ejection velocities. Recent research showed the usefulness of TVI for assessing the severity of LV and interventricular asynchrony in patients with HF receiving CRT because of the excellent time resolution which can be achieved by TVI providing frame rates of 100 frames per second (fps) or more, i.e. a time resolution of 10 ms. Another assessment of regional LV function is the calculation of the myocardial velocity gradient or strain rate imaging, reflecting deformation and thereby a more direct measurement of contraction and relaxation using data set from color-coded TVI [31-33]. Postprocessing the color coded TVI (c-TVI) data set can as well be used to evaluate the time related contraction pattern of the LV, which is less angle dependent than strain itself. Major interest of the regional time aspect of LV contraction measured bt TVI has increased because of the promising results of CRT in HF patients.

### Conventional echocardiography

#### M-Mode

150 ms  predicted response to CRT in terms of > 15 % reduction of left ventricular end-systolic volume index in 79% of the patients. The authors demonstrated the prediction of reverse remodelling by septal-to-posterior wall motion delay to be more precise than the QRS duration (accuracy 85% vs. 65%) Using the septal-to-posterior wall motion delay in M-mode recordings from the parasternal short axis view Pitzalis et al. demonstrated in 20 patients that a septal-to-posterior wall motion delay  of  ≥ 130 ms and QRS duration   ≥[34]. The same group demonstrated using this method in 60 patients that ischemic cardiomyopathy, changes in the QRS duration after implantation, and SPWMD significantly correlated with progression toward HF (defined as a worsening clinical condition leading to a sustained increase in conventional therapies, hospitalization, cardiac transplantation, and death). A long SPWMD remained significantly associated with a reduced risk of HF progression. An improvement in LVEF was observed in 79% of the patients with a baseline SPWMD of  ≥ 130 ms and in 9% of those with an SPWMD of <130 ms (p <0.0001) [35].

**Figure 1:**
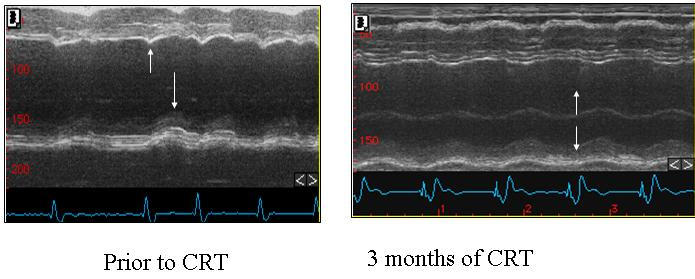
The septal-to-posterior wall motion delay in M-mode recordings can be examined in the parastrenal long axis view and a decrease of the posterior delay during CRT can be shown

The septal-to-posterior wall motion delay in M-mode recordings can also be examined in the parastrenal long axis view and a decrease of the posterior delay during CRT can be shown (Figure 1).

#### Doppler echocardiography

The measurement of interventricular electromechanical delay using pulsed Doppler imaging was examined by Rouleau  et al in 35 patients with DCM [36]. A QRS width of more than 150 ms was correlated with a delayed aortic flow compared to the pulmonary flow as well as a delayed mitral annulus systolic wave compared to tricuspid annulus systolic wave.  The authors concluded that QRS of more than 150 ms is a good marker of interventricular mechanical asynchrony.

Various echocardiographic parameters of ventricular asynchrony were examined by Bordachar et. al in 41 patients undergoing CRT. Changes in interventricular asynchrony, defined as the difference between the aortic and pulmonary pre-ejection delays and determined as the time from the onset of the QRS complex to the beginning of each respective systolic ejection by pulsed wave Doppler imaging were not correlated with changes in cardiac output, whereas several TVI modalities could confirm high correlation with hemodynamic changes [37].

The use of pulsed tissue velocity Doppler imaging by Ansalone et al in 21 non ischemic patients receiving CRT demonstrated a reduction of asynchronous contraction of LV basal segments. After CRT, LV performance improved significantly in patients with better LV resynchronization evaluated by TVI, whereas the QRS narrowing was not predictive of this functional improvement [38].

The effect of inflow based AV optimization adopted from use in DDD pacing [39] showed in CRT responders, that changes in preload only partly could explain the improved LV performance in terms of pulse pressure. The authors emphasize the importance of LV resynchronization for improved LV performance [40].

Functional mitral regurgitation is reduced by CRT in patients with HF and LBBB shown in 24 patients by Breithardt et al, explained by a more coordinated LV contraction and due to the increased closing force (left ventricular systolic pressure rise) [41].

### Newer echocardiographic methods

#### Contrast echocardiography

A new echocardiographic method based on contrast variability imaging was used in 10 patients by Kawaguchi et al. to quantify asynchrony and magnitude of resynchronization achieved by CRT [42]. The method showed that lateral wall motion occurred earlier during CRT and that both spatial and temporal asynchrony in the LV contraction declined with LV pacing and CRT correlated with increasing ejection fraction.

#### Borderline detection

A semiautomatic border detection method based on the fact that each region of the ventricular endocardial wall undergoes a periodic cycle of inward and outward displacement was performed in 34 patients by Breithardt et al. The authors showed a septal-lateral resynchronization during CRT by using this echocardiographic phase analysis of radial endocardial wall motion predicting an acute hemodynamic response (dp/dt) [43].

#### Three dimensional echocardiography

Three dimensional (3 D) echocardiography has been used by Kim et al. to document hemodynamic improvement by CRT  and additionally to identify LV segments with asynchrony [44]. The latter 3D echocardiographic method uses semi-automated contour analysis by a fast-rotating second harmonic transducer and was used in 16 patients by Krenning et al. to demonstrate a reduction of the LV contraction delay during CRT. The authors claim that this new 3D echocardiographic method might also be used to select the optimal pacing site during CRT [45].

#### Tissue Color Doppler Velocity Imaging (c-TVI)

**Figure 2:**
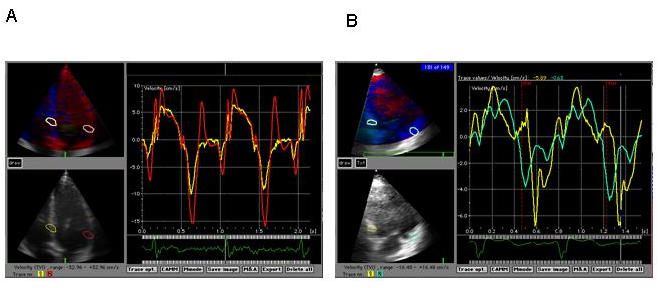
c-TVI curves at basal sites of interventricular septum (IS) and lateral free wall (LFW). **A** Control person: normal peak velocity and synchronous peaks at IS (yellow line) and LFW (red line). **B** Heart failure patient with LBBB: reduced peak velocities and asynchronous peaks at IS (yellow line) and LFW (green line).

The excellent time resolution of c-TVI by using a postprocessing procedure is used in several studies to demonstrate the regional time aspect of LV contraction. In addition to the high time resolution another main advantage of c-TVI method is the possibility of comparing the contraction pattern of different LV regions simultaneously from the recording. Synchronicity of LV contraction pattern in the structurally normal heart is demonstrated  and compared to HF patients with bundle brunch block  showing a significant asynchrony within the LV in the HF patients [46,47]. (Figure 2)

Comparing surface ECG with the mechanical contraction pattern in HF patients showed that neither QRS duration nor QRS pattern predicts the mechanical asynchrony. Even HF patients with normal QRS width can have significant LV asynchronous contraction [48,49]. The reduction of post systolic LV contraction was demonstrated during CRT by Soegard et al. and is assumed to be an indirect marker of LV resynchronization [50]. Based on data from 25 patients receiving CRT the same group did also show by tissue Doppler imaging that  occurrence of postsystolic contraction prior to CRT may predicts improved systolic performance and reversed LV remodelling during CRT  whereas QRS duration failed to predict resynchronization efficacy [51].

Using c-TVI has shown resynchronization during CRT, both as measurement of regional contraction timed to the QRS complex  by Yu et al. and measured as an absolute resynchronization of the contraction of LV septum and the lateral free wall by Schuster et al. and by Bax et al [52-54]. One study by Bax et al in 25 patients showed a possible predictive value of c-TVI asynchrony prior to implantation  which points in the same direction as Yu et al  in 30 patients [55,56].

#### Strain and strain rate imaging

The measurement of LV segment shortening or lengthening (strain) is also used to demonstrate resynchronization during CRT, but the more angle dependency of strain compared to TVI allows only the measurement of relative resynchronization because of the fact that the different regions have to be examined in separate recordings. However a resynchronization effect by CRT is shown using the strain method by Breithardt et al [57].

## Summary

The above mentioned manuscripts deal partly with concordance or disconcordance of electrical and mechanical markers of asynchrony. Despite the findings of angioscintigraphic asynchrony related to the QRS complex [58], in summary surface ECG seems not to be a sufficient marker of the mechanical events within the LV. However, the invasive endocardial activation mapping correlated well with tissue Doppler imaging to  locate the latest segment of activation in the 7 patients with HF and LBBB published by Yu et al [21]. The possibility of improving the LV function deteriorated by asynchrony using CRT, calls for a method of selecting patients with mechanical LV asynchrony prior to implantation of CRT. The published material so far demands in our opinion at least one additional method to surface ECG to reveal LV asynchrony. Several non invasive methods have been used and in small studies even predicted clinical or hemodynamic improvement by CRT. There is still no gold standard for assessment of LV asynchrony for CRT, but both traditional and new echocardiographic methods have shown both asynchronous LV contraction in HF patients, and resynchronized LV contraction during CRT and should be implemented as additional methods for selecting patients to CRT.

## References

[1] Gregoratos G, Abrams J, Epstein AE (2002). ACC/AHA/NASPE 2002 guideline update for implantation of cardiac pacemakers and antiarrhythmia devices: summary article: a report of the American College of Cardiology/American Heart Association Task Force on Practice Guidelines (ACC/AHA/NASPE Committee to Update the 1998 Pacemaker Guidelines). Circulation.

[2] Hochleitner M, Hortnagl H, Ng CK (1990). Usefullness of Physiological Dual-chamber Pacing in Drug Resistant Idiopathic Dilated Cardiomyopathy. Am J Cardiol.

[3] Gold MR, Feliciano Z, Gottlieb SS (1995). Dual-Chamber Pacing With a Short Atrioventricular Delay in Congestive Heart Failure: A Randomized Study. J Am Coll Cardiol.

[4] Cowell R, Morris-Thurgood J, Ilsley C (1994). Septal short atrioventricular delay pacing: additional hemodynamic improvements in heart failure. PACE.

[5] Gold MDP, Shorofsky MDP, Metcalf BA (1997). The Acute Hemodynamic Effects of Right Ventricular Septal Pacing in Patients With Congestive Heart Failure Secondary to Ischemic or Idiopathic Dilated Cardiomyopathy. Am J Cardiol.

[6] Victor F, Leclercq C, Mabo P (1999). Optimal right ventricular pacing site in chronically implanted patients; A prospective randomized crossover comparison of apical and outflow tract pacing. J Am Coll Cardiol.

[7] Grines CL, Bashore TM, Boudoulas H (1989). Functional abnormalities in isolated left bundle branch block. The effect of interventricular asynchrony. Circulation.

[8] Prinzen FW, Cheriex EC, Delhaas T (1995). Asymmetric thickness of the left ventricular wall resulting from asynchronous electric activation: a study in dogs with ventricular pacing and in patients with left bundle branch block.. Am Heart J.

[9] Verbeek XA, Vernooy K, Peschar M (2003). Intra-ventricular resynchronization for optimal left ventricular function during pacing in experimental left bundle branch block. J Am Coll Cardiol.

[10] Xiao HB, Brecker SJD, Gibson DG (1992). Effects of Abnormal Activation on the Time Course of the Left-Ventricular Pressure Pulse in Dilated Cardiomyopathy. Brit Heart Journal.

[11] Blanc JJ, Etienne Y, Gilard M (1997). Evaluation of different ventricular pacing sites in patients with severe heart failure: Results of an acute hemodynamic study. Circulation.

[12] Leclercq C, LeBreton H, Pavin D (1997). Acute hemodynamic response to biventricular DDD pacing in patients with severe congestive heart failure and without conventional indication for permanent pacemaker. Circulation.

[13] Kass DA, Chen CH, Curry C (1999). Improved left ventricular mechanics from acute VDD pacing in patients with dilated cardiomyopathy and ventricular conduction delay. Circulation.

[14] Nelson GS, Berger RD, Fetics BJ (2000). Left ventricular or biventricular pacing improves cardiac function at diminished energy cost in patients with dilated cardiomyopathy and left bundle-branch block. Circulation.

[15] Auricchio A, Stellbrink C, Sack S (2002). Long-term clinical effect of hemodynamically optimized cardiac resynchronization therapy in patients with heart failure and ventricular conduction delay. J Am Coll Cardiol.

[16] Abraham WT, Fisher WG, Smith AL (2002). Cardiac resynchronization in chronic heart failure. N Eng J Med.

[17] Young JB, Abraham WT, Smith AL (2003). Combined cardiac resynchronization and implantable cardioversion defibrillation in advanced chronic heart failure: the MIRACLE ICD Trial. JAMA.

[18] Baldasseroni S, Opasich C, Gorini M (2002). Left bundle-branch block is associated with increased 1-year sudden and total mortality rate in 5517 outpatients with congestive heart failure: A report from the Italian network on congestive heart failure. Am Heart J.

[19] Alonso C, Leclercq C, Victor F (1999). Electrocardiographic predictive factors of long-term clinical improvement with multisite biventricular pacing in advanced heart failure. Am J Cardiol.

[20] Peichl P, Kautzner J, Cihak R (2004). The spectrum of inter- and intraventricular conduction abnormalities in patients eligible for cardiac resynchronization therapy. PACE.

[21] Fung JWH, Yu CM, Yip G (2004). Variable left ventricular activation pattern in patients with heart failure and left bundle branch block. Heart.

[22] Lambiase PA, Rinaldi A, Hauck J (2004). Non-contact left ventricular endocardial mapping in cardiac resynchronisation therapy. Heart.

[23] Curry CW, Nelson GS, Wyman BT (2000). Mechanical dyssynchrony in dilated cardiomyopathy with intraventricular conduction delay as depicted by 3D tagged magnetic resonance imaging. Circulation.

[24] Fauchier L, Marie O, Casset-Senon D (2003). Reliability of QRS duration and morphology on surface electrocardiogram to identify ventricular dyssynchrony in patients with idiopathic dilated cardiomyopathy. Am J Cardiol.

[25] Toussaint JF, Lavergne T, Ollitraut J (2000). Biventricular pacing in severe heart failure patients reverses electromechanical dyssynchronization from apex to base. PACE.

[26] Toussaint JF, Lavergne T, Kerrou K (2003). Basal asynchrony and resynchronization with biventricular pacing predict long-term improvement of LV function in heart failure patients. PACE.

[27] Kerwin WF, Botvinick EH, O'Connell JW (2000). Ventricular contraction abnormalities in dilated cardiomyopathy: effect of biventricular pacing to correct interventricular dyssynchrony. J Am Coll Cardiol.

[28] Schiller NB, Shah PM, Crawford M (1989). Recommendations for quantitation of the left ventricle by two-dimensional echocardiography. American Society of Echocardiography Committee on Standards, Subcommittee on Quantitation of Two-Dimensional Echocardiograms. J Am Soc Echocardiogr.

[29] Gorcsan J, Strum DP, Mandarino WA (1997). Quantitative assessment of alterations in regional left ventricular contractility with color-coded tissue Doppler echocardiography - Comparison with sonomicrometry and pressure-volume relations. Circulation.

[30] Zamorano J, Wallbridge DR, Ge J (1997). Non-invasive assessment of cardiac physiology by tissue Doppler echocardiography - A comparison with invasive haemodynamics. Eur Heart J.

[31] Heimdal A, Stoylen A, Torp H (1998). Real-time strain rate imaging of the left ventricle by ultrasound. J.Am.Soc.Echocardiogr.

[32] Urheim S, Edvardsen T, Torp H (2000). Validation of a new method to quantify regional myocardial function. Circulation.

[33] Edvardsen T, Gerber BL, Garot J (2002). Quantitative assessment of intrinsic regional myocardial deformation by Doppler strain rate echocardiography in humans: validation against three-dimensional tagged magnetic resonance imaging. Circulation.

[34] Pitzalis MV, Iacoviello M, Romito R (2002). Cardiac resynchronization therapy tailored by echocardiographic evaluation of ventricular asynchrony. J Am Coll Cardiol.

[35] Pitzalis MV, Iacoviello M, Romito R (2005). Ventricular asynchrony predicts a better outcome in patients with chronic heart failure receiving cardiac resynchronization therapy. J Am Coll Cardiol.

[36] Rouleau FDR, Merheb M, Geffroy S (2001). Echocardiographic assessment of the interventricular delay of activation and correlation to the QRS width in dilated cardiomyopathy. PACE.

[37] Bordachar P, Lafitte S, Reuter S (2004). Echocardiographic parameters of ventricular dyssynchrony validation in patients with heart failure using sequential biventricular pacing. J Am Coll Cardiol.

[38] Ansalone G, Giannantoni P, Ricci R (2001). Adrenergic nervous system activity in patients after surgical correction of tetralogy of Fallot. Am Heart J.

[39] Kindermann M, Frohlig G, Doerr T (1997). Optimizing the AV delay in DDD pacemaker patients with high degree AV block: mitral valve Doppler versus impedance cardiography. Pacing Clin Electrophysiol.

[40] Auricchio AF, Ding J, Spinelli JC (2002). Cardiac resynchronization therapy restores optimal atrioventricular mechanical timing in heart failure patients with ventricular conduction delay. J Am Coll Cardiol.

[41] Breithardt OA, Sinha AM, Schwammenthal E (2003). Acute effects of cardiac resynchronization therapy on functional mitral regurgitation in advanced systolic heart failure. J Am Coll Cardiol.

[42] Kawaguchi M, Murabayashi T, Fetics BJ (2002). Quantitation of basal dyssynchrony and acute resynchronization from left or biventricular pacing by novel echo-contrast variability imaging. J Am Coll Cardiol.

[43] Breithardt OA, Stellbrink C, Kramer AP (2002). Echocardiographic quantification of left ventricular asynchrony predicts an acute hemodynamic benefit of cardiac resynchronization therapy. J Am Coll Cardiol.

[44] Kim WY, Sogaard RA, Mortensen PT (2001). Three dimensional echocardiography documents haemodynamic improvement by biventricular pacing in patients with severe heart failure. Heart.

[45] Krenning BJ, Szili-Torok T, Voormolen MM (2004). Guiding and optimization of resynchronization therapy with dynamic three-dimensional echocardiography and segmental volume--time curves: a feasibility study. Eur J Heart Fail.

[46] Schuster P, Faerstrand S, Ohm O (2004). Feasability of color tissue velocity imaging for assesment of regional timing of left ventricular longitudinal movement. Scand Cardiovasc J.

[47] Schuster P, Faerestrand S, Ohm OJ (2004). Color Doppler Tissue Velocity Imaging Demonstrates Significant Asynchronous Regional Left Ventricular Contraction and Relaxation in Patients With Bundle Branch Block and Heart Failure Compared to Control Subjects. Cardiology.

[48] Yu CM, Yang H, Lau CP (2003). Regional left ventricle mechanical asynchrony in patients with heart disease and normal QRS duration: implication for biventricular pacing therapy. PACE.

[49] Schuster P, Faerestrand S, Ohm OJ (2004). Color Doppler Tissue Velocity Imaging Can Disclose Systolic Left Ventricular Asynchrony Independent of the QRS Morphology in Patients with Severe Heart Failure. PACE.

[50] Sogaard P, Kim WY, Jensen HK (2001). Impact of acute biventricular pacing on left ventricular performance and volumes in patients with severe heart failure - A tissue Doppler and three-dimensional echocardiographic study. Cardiology.

[51] Sogaard P, Egeblad H, Kim WY (2002). Tissue doppler imaging predicts improved systolic performance and reversed left ventricular remodeling during long-term cardiac resynchronization therapy. J Am Coll Cardiol.

[52] Yu CM, Chau E, Sanderson JE (2002). Tissue Doppler echocardiographic evidence of reverse remodeling and improved synchronicity by simultaneously delaying regional contraction after biventricular pacing therapy in heart failure. Circulation.

[53] Bax JJ, Molhoek SG, van Erven L (2003). Usefulness of myocardial tissue Doppler echocardiography to evaluate left ventricular dyssynchrony before and after biventricular pacing in patients with idiopathic dilated cardiomyopathy. Am J Cardiol.

[54] Schuster P, Faerestrand S, Ohm OJ (2003). Colour tissue velocity imaging can show resynchronisation of longitudinal left ventricular contraction pattern by biventricular pacing in patients with severe heart failure. Heart.

[55] Bax JJ, Marwick TH, Molhoek SG (2003). Left ventricular dyssynchrony predicts benefit of cardiac resynchronization therapy in patients with end-stage heart failure before pacemaker implantation. Am J Cardiol.

[56] Yu CM, Fung WH, Lin H (2003). Predictors of left ventricular reverse remodeling after cardiac resynchronization therapy for heart failure secondary to idiopathic dilated or ischemic cardiomyopathy. Am J Cardiol.

[57] Breithardt OA, Stellbrink C, Herbots L (2003). Cardiac resynchronization therapy can reverse abnormal myocardial strain distribution in patients with heart failure and left bundle branch block. J Am Coll Cardiol.

[58] Toussaint JF, Lavergne T, Kerrou K (2002). Ventricular coupling of electrical and mechanical dyssynchronization in heart failure patients. PACE.

